# Fickle or Faithful: The Roles of Host and Environmental Context in Determining Symbiont Composition in Two Bathymodioline Mussels

**DOI:** 10.1371/journal.pone.0144307

**Published:** 2015-12-28

**Authors:** Sven R. Laming, Kamil M. Szafranski, Clara F. Rodrigues, Sylvie M. Gaudron, Marina R. Cunha, Ana Hilário, Nadine Le Bris, Sébastien Duperron

**Affiliations:** 1 Sorbonne Universités, UPMC Université Paris 06, UMR7208 Laboratoire biologie des organismes et écosystèmes aquatiques (UPMC CNRS MNHM IRD CAEN), 7 quai St Bernard, Paris, France; 2 Departamento de Biologia and CESAM, Universidade de Aveiro, Campus Universitario de Santiago, Aveiro, Portugal; 3 Sorbonne Universités, UPMC Banyuls, UMR8222 Laboratoire d'Ecogéochimie des Environnements Benthiques (UPMC CNRS), Station marine de Banyuls, Observatoire Océanologique, Banyuls-sur-Mer, France; 4 Institut Universitaire de France, Paris, France; Australian Museum, AUSTRALIA

## Abstract

The Mediterranean Sea and adjoining East Atlantic Ocean host a diverse array of small-sized mussels that predominantly live on sunken, decomposing organic remains. At least two of these, *Idas modiolaeformis* and *Idas simpsoni*, are known to engage in gill-associated symbioses; however, the composition, diversity and variability of these symbioses with changing habitat and location is poorly defined. The current study presents bacterial symbiont assemblage data, derived from 454 pyrosequencing carried out on replicate specimens of these two host species, collected across seven sample sites found in three oceanographic regions in the Mediterranean and East Atlantic. The presence of several bacterial OTUs in both the Mediterranean Sea and eastern Atlantic suggests that similar symbiont candidates occur on both sides of the Strait of Gibraltar. The results reveal markedly different symbiotic modes in the two species. *Idas modiolaeformis* displays high symbiont diversity and flexibility, with strong variation in symbiont composition from the East Mediterranean to the East Atlantic. *Idas simpsoni* displays low symbiont diversity but high symbiont fidelity, with a single dominant OTU occurring in all specimens analysed. These differences are argued to be a function of the host species, where subtle differences in host evolution, life-history and behaviour could partially explain the observed patterns. The variability in symbiont compositions, particularly in *Idas modiolaeformis*, is thought to be a function of the nature, context and location of the habitat from which symbiont candidates are sourced.

## Introduction

Communities of chemosynthetic prokaryotes are crucial in sustaining the high densities of specialist Metazoa occurring in marine habitats enriched in reduced chemical compounds, particularly through symbioses [1]. Reducing conditions in the deep sea can occur at hydrothermal vents, hydrocarbon seeps and organic-fall habitats. The mytilid subfamily Bathymodiolinae has colonised nearly all reducing habitats in the deep sea at some point during its evolution [2–4]. Its prominence at vents and seeps is well-documented, the genus *Bathymodiolus* being one of the most ubiquitous and abundant genera recorded at these habitats globally [5]. However, the ecological importance of several clades of much smaller-sized bathymodioline species (maximum shell length, SL_max_ <5cm, [6]) has since been highlighted by their prevalence on decomposing whale carcasses and sunken plant debris. The subfamily’s overall success is attributed to adaptive metabolic capabilities provided by gill-associated chemosynthetic bacteria [1], present in nearly all adult bathymodioline species investigated anatomically to date. These symbioses most frequently involve chemolithoautotrophic bacteria, often capable of sulphur-oxidation (SOX), or methylotrophic bacteria (most being methane-oxidisers, MOX). Certain species can host dual or even multiple symbioses (e.g. [7]).

When compared with larger seep and vent species, smaller bathymodiolins can often colonise a greater diversity of habitats over a broader geographical scale [8], particularly in the Mediterranean Sea and adjoining Iberian margin where they are prevalent on various organic falls and cold seeps ([5], and references therein). In at least one small-sized bathymodioline that hosts a variety of symbionts, *Idas modiolaeformis*, bacterial composition and diversity appears to vary with collection site, revealing a level of variability not yet reported in larger vent and seep mussels [9]. This species is recorded from three adjoining oceanographic regions: the East and West Mediterranean, and the East Atlantic [9–11] separated by the Straits of Sicily and Gibraltar, respectively. To date, more than 10 different 16S rRNA-encoding bacterial gene sequences have been recorded in association with *I*. *modiolaeformis* living on sunken wood or at methane seeps across its known geographical range [7,9,12]. Of these, between 1 and 6 bacterial lineages forming distinct 16S rRNA phylotypes have been documented in any one adult specimen. Symbiont diversity and density in *I*. *modiolaeformis* could depend on a variety of factors including habitat characteristics and locality [9,12]. Symbioses in small-sized mussels from these regions have only been documented in one other species, *Idas simpsoni* (e.g. [13,14]). Despite similar sizes and overall aspect however, studies suggest these two species should be classified in distinct genera [6]. Like *I*. *modiolaeformis*, this species’ habitat-use and geographic range are extensive, occurring in the East and West Mediterranean Sea, the southern Iberian margin and the North Sea [11], and colonising sunken wood ([11]; this study), oily drill cuttings [13] and at least one cold-seep site [15] (species confirmation in [14]). However, it is on the bones of sunken mammal carcasses that this species reaches its most prolific densities (e.g. [14,16,17]). Even though *I*. *simpsoni* records extend farther afield and occur on a greater variety of substrates than those of *I*. *modiolaeformis*, symbioses described in *I*. *simpsoni* to date have only confirmed a single, extracellular gill-associated thiotrophic bacterium [13–15].

Despite symbiont diversity having been investigated in more detail in these species than most small bathymodiolins, the degree to which observed symbiont patterns in each species are representative remains indeterminate. Prior study methods differed in their capacity to detect and quantify symbiont densities (e.g. [7] vs [9]), with limited specimen availability often preventing the evaluation of inherent, intra-site variability in symbiont compositions. Given the changeable, unexpectedly high symbiont diversity of *I*. *modiolaeformis* and the limited data available for *I*. *simpsoni*, better documenting the full diversity of symbionts is mandatory. High-throughput sequencing allows us to thoroughly assess the composition and diversity of symbiont assemblages in individual hosts. Though not strictly quantitative it allows within-study comparisons of abundance at several scales, assuming appropriate sample replication [18]. This type of approach has proven powerful as a tool to study symbioses in a variety of host organisms from terrestrial (e.g. [19,20]) and marine environments (e.g. Vesicomyidae, [21]; several marine Porifera, [22]). The symbiotic life-history characteristics of *I*. *modiolaeformis* and *I*. *simpsoni* and their occurrence on either side of both the Straits of Gibraltar and Sicily, make them ideal model species for comparative research into symbiosis.

In the current study, replicate specimens of *I*. *modiolaeformis* and *I*. *simpsoni* were collected from 7 sites in the Mediterranean Sea and the East Atlantic, and the diversity and composition of symbiotic bacterial OTUs (Operational Taxonomic Units) was comprehensively evaluated by 454-pyrosequencing the hyper-variable, V5-V6 regions of 16S rRNA-encoding genes in the gill-associated bacteria. The study sought to test whether patterns in symbiont composition and diversity are environmentally constrained in smaller-sized bathymodiolins (e.g. by host type, habitat type and locality); whether increased symbiont diversities afford small-sized bathymodiolins a greater diversity of useable habitats, and which are the factors most likely to determine host-dependent variability in symbiont assemblages.

## Materials and Methods

### Sampling and processing

**Fig 1:**
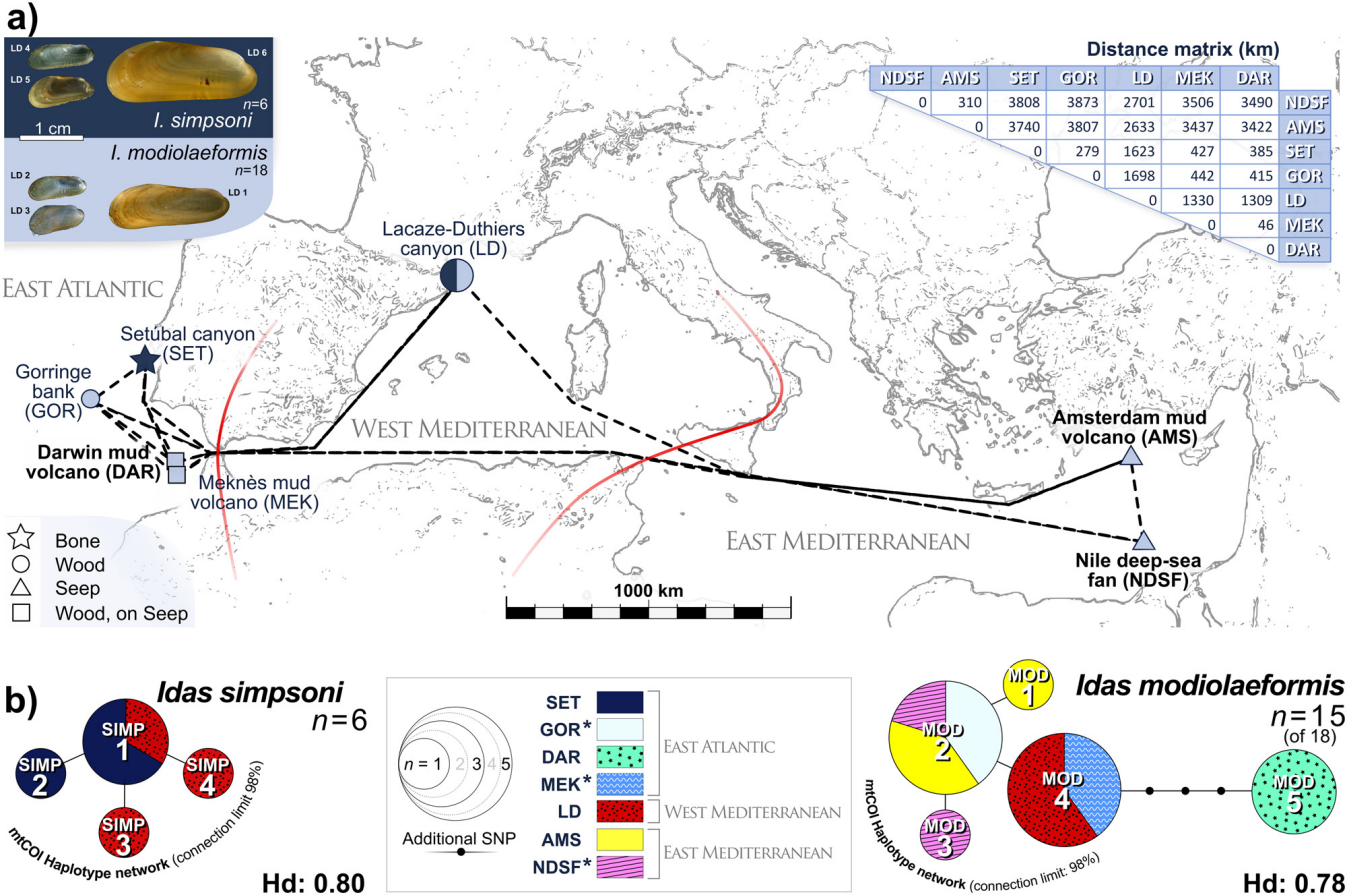
Map of the study region and mitochondrial-COI haplotype distribution. a) Presence/absence of *Idas simpsoni* (navy) and *I*. *modiolaeformis* (pale blue), habitat type and inter-site distances are depicted for the 7 sites, which span three oceanographic regions (demarcated by red lines). Sites where seepage was visibly active (e.g. turbulent flow / bubbling from sediments) are labelled in bold black. Distance matrix relates to dashed-line trajectories, measured in Google Earth^™^. Example micrographs of both hosts (top-left) are specimens LD 1–6, from LD. b) The mtCOI haplotype networks for *I*. *simpsoni* and *I*. *modiolaeformis* are displayed (see central legend). Sequences of adjacent haplotypes differed by a single nucleotide polymorphism (SNP). Diameters represent overall haplotype frequencies, subdivided by site location, where applicable. Hd- Haplotype diversity; 1 SNP = 0.4% K2P distance (257bp). Asterisks indicate sites where COI was analysed in 2 of 3 hosts only. For sample/site details, see Table 1 and main text. Base map redrawn from a Google Earth^™^ image.

Specimens of *Idas modiolaeformis* and *I*. *simpsoni* were retrieved during cruises from 2007–2013 when sampling a variety of substrates and habitats at seven sites (Table 1) from three oceanographic regions: the East and West Mediterranean and the East Atlantic (Fig 1, red borders delineate the straits of Sicily and Gibraltar between each region). To assess symbiont assemblages, gill tissue was analysed from three replicate mussels per species, per site (*n* = 24). Most specimens had colonised plant substrate either found *in situ* (Gorringe Bank [GOR], southern Iberian margin, East Atlantic), or deployed experimentally either as a section of palm trunk (Lacaze-Duthiers canyon [LD], Gulf of Lion, West Mediterranean), or as pieces of pinewood or alfalfa grass in CHEMECOLI colonisation devices (Darwin [DAR] and Meknès [MEK] mud volcanoes in the Gulf of Cadiz, East Atlantic; and LD; Table 1; see [23] for construction). Remaining specimens were either attached to sunken-bone remnants from whole-carcass deployments (Setúbal canyon, southern Iberian margin, East Atlantic [SET]; see [14] for experimental details), or collected directly from carbonate crusts at two East Mediterranean methane seeps (Amsterdam mud volcano [AMS]; Nile deep-sea fan [NDSF]). Fixation (Table 1) was in 96% ethanol (4°C storage), except for samples from LD, which were flash-frozen in liquid nitrogen (-80°C storage). Of the four seep-like sites (i.e. ‘Seep’, and ‘Wood, on Seep’), visible activity was discernible at three: NDSF, AMS, and DAR (Table 1). This study on mollusc bivalves did not involve endangered species, sites were not privately owned and, with the exception of LD, were not from protected areas. Authorisation to deploy experiments in LD (located in the Gulf of Lion Marine Park) was obtained in advance (NLB). No specific permission was required for the sampling of *Idas* specimens.

**Table 1:** Cruise, site and sampling details for substrates on which the two target host mussel species were found.

mtCOI Haplotype	Replicates	Acc. number	Cruise (yr)	Site	Coordinates	Habitat	Depth (m)	SL (mm)	Deployment (wks)	*Ref*.
***Idas modiolaeformis***										
MOD 1	AMS 1	KT216492	MERIAN (2009)	**Amsterdam MV**	**N** 35° 20.079	Seep	2031	11.4	-	[12,24]
MOD 2	AMS 2	KT216493		*East Mediterranean*	**E** 30° 16.131			10.2		
MOD 2	AMS 3	KT216494						8.4		
MOD 3	NDSF 1	KT216495	MEDECO (2007)	**Nile deep-sea fan**	**N** 32° 30.030	Seep^1^	1686	6.6	-	[12,24]
MOD 2	NDSF 2	KT216496		*East Mediterranean*	**E** 30° 15.604			6.8		
-	NDSF 3	-						8.1		
MOD 5	DAR 1	KT216482	B09-14b (2009)	**Darwin mud volcano**	**N** 35° 23.523	CHEMECOLI^2^	1100	-	104	[25]
MOD 5	DAR 2	KT216483		*East Atlantic*	**W** 07° 11.513			-		
MOD 5	DAR 3	KT216484						-		
MOD 4	MEK 1	KT216485	64PE284 (2009)	Meknès mud volcano	**N** 34° 59.09	CHEMECOLI^3^	698	-	61	[25]
MOD 4	MEK 2	KT216486		*East Atlantic*	**W** 07° 04.42			-		
-	MEK 3	-				CHEMECOLI^2^		-		
MOD 2	GOR 1	KT216487	NA017 (2011)	Gorringe Bank	**N** 36° 38.557	Unknown	1308	-	-	[25]
MOD 2	GOR 2	KT216488		*East Atlantic*	**W** 11° 36.187	wood type		-		
-	GOR 3	-						-		
MOD 4	LD 1	KT216497	Banyuls #LD5 (2013)	Lacaze-Duthiers canyon	**N** 42° 32.728	CHEMECOLI^2^	525	8.9	42	[26]
MOD 4	LD 2	KT216499		*West Mediterranean*	**E** 03° 25.267	Palmwood	525	9.4	129	[26]
MOD 4	LD 3	KT216500						19.5		
***Idas simpsoni***										
SIMP 1	LD 4	KT216498	Banyuls #LD5 (2013)	Lacaze-Duthiers canyon	**N** 42° 32.728	Palmwood	525	10.6	129	[26]
SIMP 3	LD 5	KT216501		*West Mediterranean*	**E** 03° 25.267			10.7		
SIMP 4*	LD 6 *	KT216502*						24.2 *		
SIMP 1	SET 1	KT216489	CARCACE (2012)	Setúbal Canyon	**N** 38° 16.85	Bone remains	1000	-	78	[14]
SIMP 1	SET 2	KT216490		*East Atlantic*	**W** 09° 06.68	(cow carcass)		-		
SIMP 2	SET 3	KT216491						-		

CHEMECOLI refers to the use of colonisation devices for CHEMosynthetic Ecosystem COlonization by Larval Invertebrates. For details regarding haplotypes, see Fig 1b. Visibly active sites are in bold. Habitat superscripts: **1** Carbonate crust present; **2** Pinewood cubes; **3** Packed alfalfa substrate.

* Specimen discarded (see main text).

### Host identification and mitochondrial COI haplotypes

Total DNA was extracted from gill tissue using the QIAamp^®^ DNeasy blood and tissue kit (QIAGEN, USA). Host identification was based on the mitochondrial Cytochrome Oxidase I-encoding gene (mtCOI) and supported by morphological observations. A fragment of host mtCOI was PCR-amplified using primers LCO1490 [27] and H691 [7]. The program started at 94°C for 4 min, followed by 35 cycles of 94°C for 40 s, 50°C for 50 s, and 72°C for 1 min, with a final elongation step at 72°C for 10 min. PCR products were sequenced in both directions (GATC Biotech). Sequences were assembled and checked using BioEdit v. 7.2.5 [28]. Only regions for which all residues had been identified were used (360–501 bp, depending on individual). Sequences were queried against the GenBank nucleotide sequence database using BLAST [29]. Distinguishing morphology was limited to a more tanned, glossy periostracum in large adult *Idas simpsoni* and the presence of numerous byssal hairs on the periostraca of *I*. *modiolaeformis*, rarely found on *I*. *simpsoni* (Fig 1a).

Host mtCOI sequences with >98% sequence identity were considered to belong to the same species. Haplotype maps were compiled from haplotype networks generated in TCS v.1.21 [30] from aligned mtCOI sequence data and the geographical distribution of identified haplotypes (Table 1). Aligned sequences contained no gaps, having been edited and trimmed to 257 bp. Haplotypes and single nucleotide polymorphisms (SNPs) identified in TCS were cross-checked in DnaSP v.5.10.01 [31]. Pairwise distances were calculated from aligned sequence data for each host species in MEGA6 (Kimura 2-parameter (K2P) model, [32]). Host mtCOI sequences have been deposited in Genbank under accession numbers reported in Table 1.

### Sequencing, sequence treatment, and analyses of partial bacterial 16S rRNA

The V5-V6 variable region corresponding to region 770–1094 of the *Escherichia coli* 16S rRNA-encoding gene was amplified using primers V5V6_F (5’-CAAACAGGATTAGATACCCTG-3’) and V5V6_R (5’-CGTTRCGGGACTTAACCCAACA-3’), following the procedure in [33]. PCR products were purified and quantified by Picogreen. Specimen-specific 10-bp molecular identifier (MID) tags were inserted between the GS-FLX adapters and the specific primers to facilitate further sequence binning. An equal proportion of amplicons were added for each specimen. Short pyrotags (~300bp) were then sequenced by 454 pyrosequencing (GS-FLX Roche Diagnostics, GENOSCREEN, France) on a single slide. Only reads from between 50–450 bp with no mismatches in their MIDs were retained (85376 valid 16S partial sequences were obtained from the 24 mussel samples).

Resulting binary ‘.sff’ files were extracted using Mothur [34]. Sequences shorter than 250 bp, longer than 350 bp and containing N's were eliminated from further analysis. Filtered sequences were sorted by their MID sequences into separate Fasta files. Typical 454 sequencing errors and PCR single-base errors were screened using the PyroNoise and SeqNoise modules in AmpliconNoise [35] with default parameters. Having removed MIDs and primer nucleotides, sequences were used to generate a Needleman-Wunsch distance matrix and clustered into operational taxonomic units (OTUs) in AmpliconNoise (NDist and Fcluster functions). The matrix of sequence abundances per OTU was generated using Python scripts with a 99% identity threshold for OTU definition, resulting in 525 OTUs. The most common of the within-OTU sequences was considered representative (≈300 bp). Using BLAST [29], the four best hits per OTU (max. e-value = 1e^-10^) were identified from the SSURef_NR99_115 Silva database [36].

The complete dataset (‘total’ OTUs or assemblages) was reduced in size by removing all OTUs for which read counts were consistently below 1% of total reads in all host individuals. Those that were kept therefore only included OTUs where reads accounted for at least 1% of any one individual mussel’s bacterial assemblage (*n* = 28, described as ‘principal’ OTUs or assemblages). Those with >99% sequence identity with documented chemosynthetic bacteria or published symbionts were considered to be putative symbionts (‘symbiont’ OTUs or assemblages). Symbiont lineages were placed in context by constructing a rooted phylogenetic tree using V5-V6 sequences from this study, those of known symbiotic bacteria in marine bivalves, and, where informative, those free-living in the marine environment. Sequences were assembled, aligned (ClustalW [37]), and checked in BioEdit v.7.2.5 [28]). Trees based on maximum likelihood were constructed in MEGA6 [38]. Symbiont and environmental 16S bacterial sequences have been placed in Genbank under accession numbers KT216459 to KT216481 (detail in S1 Table).

### Symbiont-community analyses

Symbiont diversity was analysed using the Shannon-Weiner index derived from abundance data (reads OTU^-1^ host^-1^ based on principal OTUs), which integrates species richness and evenness into a single index. Sequencing bias in the data was assessed further by correlating both the number of principal OTUs and the number of symbiont OTUs against the corresponding number of principal reads. Abundance data were then log(*x*+1)-transformed prior to constructing a Bray-Curtis dissimilarity matrix (BC). BC dissimilarities were visualised by Non-metric Multidimensional Scaling (nMDS), performed with metaMDS (MASS package); nMDS goodness of fit was assessed using Shepard (Stress- and fit-based R^2^ in MASS package). Ellipses of average-linkage height were superimposed on the nMDS for comparison, derived from a BC-derived clustering dendrogram using hclust (Unweighted Pair-Group Method with arithmetic Average, UPGMA).

Four factors were used to group host mussels and associated bacterial assemblages. These were: the host species of origin (HOST, *n* = 2); the collection site for the host (SITE, *n* = 7); the environmental context of the host (HABITAT, *n* = 4), and; a combined grouping factor with two levels, host and site (HOST + SITE, *n* = 8). HABITAT included: whale-fall-like communities on bone (Bone), sunken-wood communities on plant debris undergoing decomposition (Wood), seep-like communities at sites with suspected methane seepage (Seep), and Wood-like communities within Seep-like sites (Wood, on Seep).

Global differences in the overall BC as a function of host species, site, and environmental context were assessed for statistical significance using ANOSIM (Vegan3d package) with HOST, SITE and HABITAT as the three levels for the grouping factor. Applying the same factor and levels, accumulative percentage contributions of individual OTUs to overall BC dissimilarities were determined using SIMPER (Vegan3d package), as a proxy for OTU dominance. Patterns of symbiont association in *Idas modiolaeformis* across sites were assessed further, as a function of physical separation (km) using a Mantel Test, by comparing a geographic-distance matrix to the BC for *I*. *modiolaeformis* only. Geographic distances were the shortest, linear, inter-site trajectories, avoiding landmasses, measured between GPS coordinates using the ruler function in Google Earth^™^ (Fig 1a). Given that *I*. *simpsoni* only occurred at two sites, a similar Mantel test was not performed for this species. All analyses were performed in the R environment [39]. Mean values are ± standard deviation.

## Results

### Distribution of host species and associated mtCOI haplotypes

The two host species *Idas modiolaeformis* and *I*. *simpsoni* co-occurred at only one site, LD, in the West Mediterranean, on experimental palmwood deployments (Fig 1). Aside from LD, *I*. *modiolaeformis* was also collected from AMS and NDSF in the East Mediterranean and DAR, MEK and GOR in the East Atlantic, accounting for 18 of the 24 specimens investigated and living within sunken-wood communities (Wood), seep-like communities (Seep), and at sites with characteristics of both environmental contexts (Wood, on Seep). Five distinct host mtCOI haplotypes were identified in *I*. *modiolaeformis*, the two most numerous occurring either side of the Strait of Gibraltar (MOD 2, MOD 4, Fig 1b). MOD 2 was found at both East Mediterranean seep sites (AMS, NDSF) and >3800 km away on naturally occurring sunken wood at the East Atlantic site GOR (Distance matrix, Fig 1a), while MOD 4 occurred at both the West Mediterranean site LD and the Atlantic site MEK (Fig 1b). *I*. *simpsoni* in the current study accounted for the remaining 6 specimens. In addition to those at LD (West Mediterranean), 3 specimens were recovered from bone remains at the East Atlantic site SET (Table 1, Fig 1). Four distinct host mtCOI haplotypes were identified in *I*. *simpsoni* (Fig 1b), in which SIMP 1 was recorded at both LD and SET. In each species, haplotypes typically differed by 1–2 SNPs (K2P = 0.4–0.8%) except for *I*. *modiolaeformis* MOD 5 (4–6 SNPs, K2P 2–2.4%), the haplotype of all three specimens collected at DAR, exclusively.

### Composition and diversity of bacterial assemblages by host species and site

#### OTU Composition

**Fig 2:**
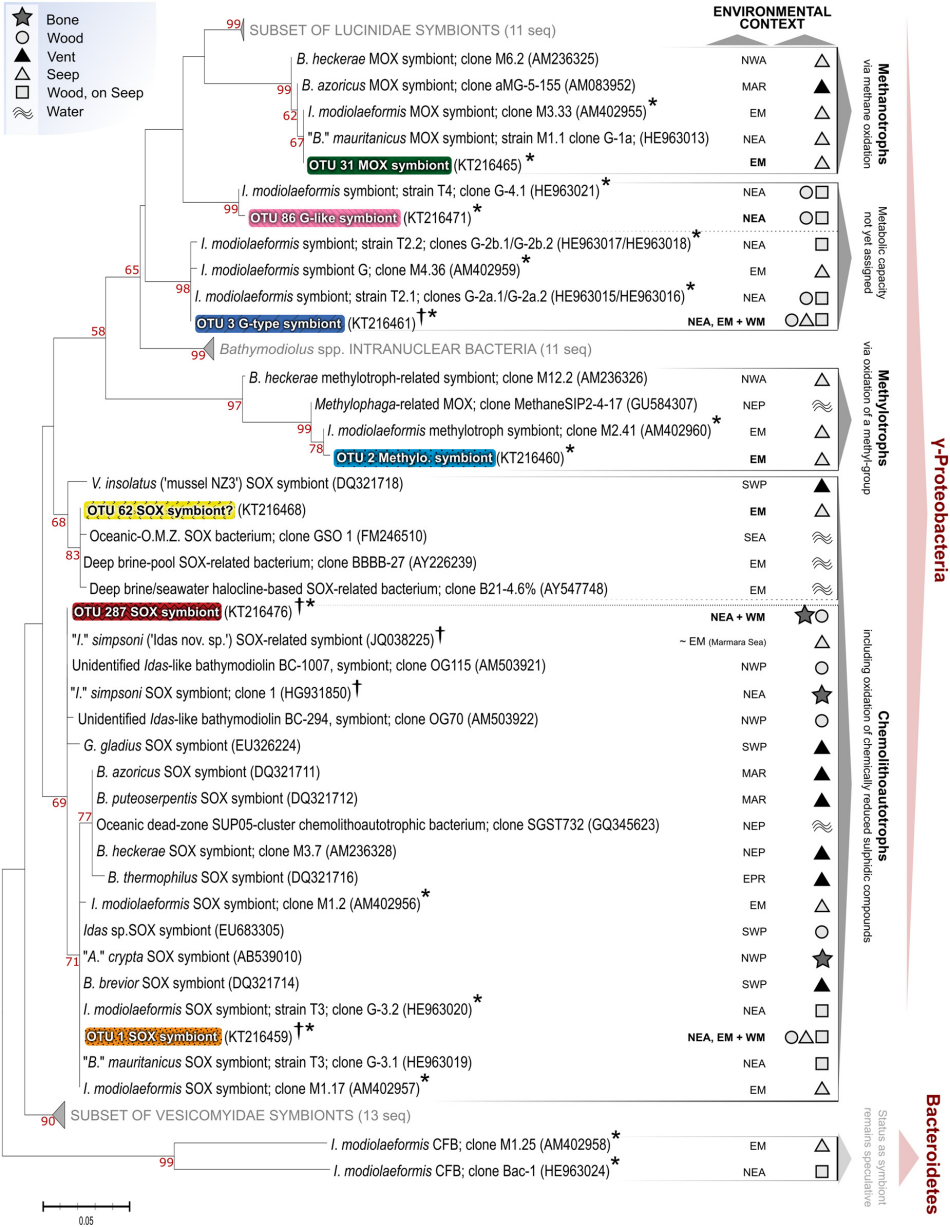
Phylogeny of symbiotic OTUs (16S rRNA) in the context of related chemosynthetic bacteria. Tree is based on partial sequences from the hypervariable V5-V6 region of 16S rRNA encoding-gene. Collapsed nodes of other chemosymbiotic bivalves only contain a subset of published symbionts, for clarity. Tree is rooted to the Bacteroidetes symbionts found in *Idas modiolaeformis* previously but not in the current study. OTU colour coding corresponds to Fig 3. Environmental context relates to corresponding sequence records, not the host species in general (EM- East Mediterranean Sea; EPR- East Pacific Rise; MAR- Mid-Atlantic Ridge; NEA- Northeast Atlantic; NEP- Northeast Pacific; NWA- Northwest Atlantic; NWP- Northwest Pacific; SEA- Southeast Atlantic; SWP- Southwest Pacific; WM- West Mediterranean; see legend for habitat). *A*.- *Adipicola*; *B*.- *Bathymodiolus*; *I*.- *Idas*; *G*.- *Gladius*; *V*.- *Vulcanidas*. Dagger- and asterisk-marked sequences are the known symbiont sequences from *I*. *simpsoni* and *I*. *modiolaeformis* respectively (includes this study).

Of the 28 principal OTUs obtained (62200 principal-OTU reads in 24 mussels), 7 were categorised as potential symbionts, displaying >99% sequence identity with known bathymodioline symbionts (Gammaproteobacteria, often SOX or MOX) or with other chemolithoautotrophic or methylotrophic Gammaproteobacteria (Fig 2). OTUs 1, 2, 3, 31 and 86 were related to known symbionts in *Idas modiolaeformis* from both the East-Mediterranean and East-Atlantic (Fig 2). OTU 1 corresponded to common chemolithautotrophic SOX bacteria of *I*. *modiolaeformis* (e.g. ‘clone M1.17’ in Fig 2) and *“B*.*” mauritanicus* (clone G 3.1). OTU 3 corresponded to the symbiont clade ‘symbiont G’ with undefined symbiotic roles, found previously in *I*. *modiolaeformis* from the East Atlantic and East Mediterranean (‘clones G-2a/G-2b’ and ‘clone M4.36’, respectively, Fig 2). OTU 2 corresponded to a methylotrophic bacterium found previously in *I*. *modiolaeformis* from the East Mediterranean (related to ‘clone M2.41’, Fig 2). OTU 86 closely matched the sequence of a symbiont-G-related bacterium identified previously in *I*. *modiolaeformis* from the East Atlantic (‘clone G-4’, Fig 2). OTU 31 was identical to a MOX symbiont known to occur in *I*. *modiolaeformis* in the East Mediterranean (‘clone M3.33’, Fig 2). OTU 287 corresponded to the one previously recorded SOX symbiont of *I*. *simpsoni* at seeps (‘*Idas*. nov. sp. SOX-related symbiont’, Fig 2) and from bones (*I*. *simpsoni* ‘clone 1’, Fig 2). The 7^th^ potential symbiont (OTU 62, recorded once in *I*. *modiolaeformis* in the current study) branched with several chemolithoautotrophic bacteria from hypersaline brine pools and an upwelling oxygen minimum zone (OMZ), the latter with confirmed SOX capabilities (‘clone GSO 1’, Fig 2). However, this bacterium matched no known marine symbiont based on its BLAST hits, and so must remain putative. The other 21 ‘non-symbiont’ OTUs recorded were either related to environmental marine bacteria, or displayed identities too ambiguous to make phylogenetic assertions (listed in S1 Table). One mussel (*I*. *simpsoni*, LD 6) harboured very different principal OTUs in comparison with the other 23 mussels. Only 3 reads of the symbiont OTU 3 were recorded. The five remaining principal OTUs identified, exclusive to this specimen, were not known symbionts and were dominated by a *Vibrio*-like sequence (88% of sequences obtained, versus 0.6% for OTU 3). LD 6 was assumed to be compromised physiologically, so this specimen was not included in further analyses.

#### OTU occurrences and symbiont diversity

**Fig 3:**
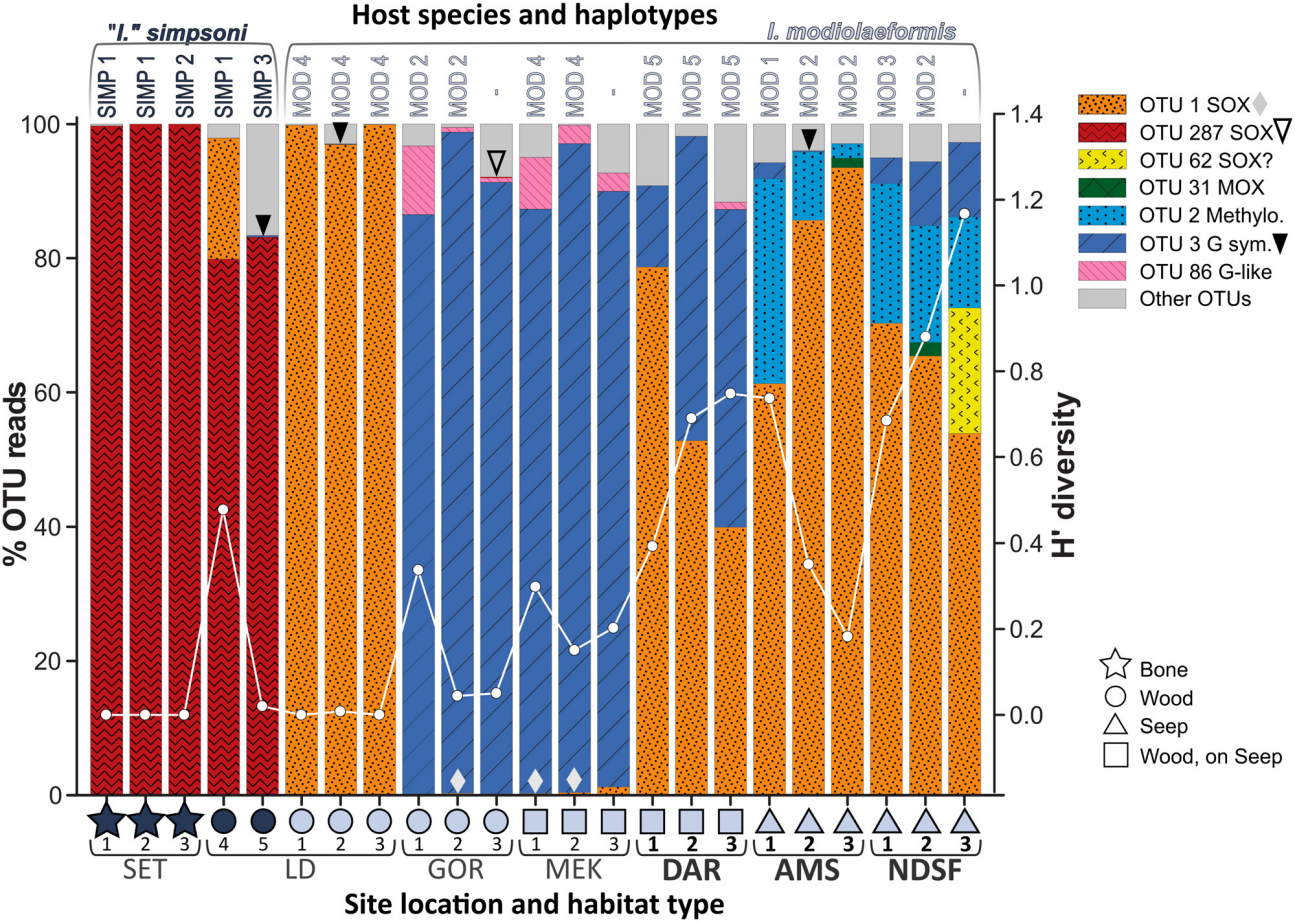
Percentage contributions of principal OTUs and the Shannon-Weiner diversity index (H’). Corresponding number of reads for principal OTUs available in S1 Table. Diamonds (bottom) and arrowheads (top) identify lowest abundances not visible as bars (corresponding OTUs in legend). Overlaid line plot is H’ diversity of symbionts. Haplotypes (above) and site details (below, see legend) are indicated (N.B. COI in 3 of 18 *Idas modiolaeformis* not analysed). Visibly active sites are in bold. Analyses exclude mussel LD 6, which was probably moribund (see main text).

The mean number of principal-OTU reads specimen^-1^ was 2682 ±992. Both host species were dominated numerically by symbiont-related OTUs (83–100% of principal-OTU reads identified specimen^-1^, Fig 3, S1 Table). The number of distinct symbiont OTUs occurring in a single host specimen varied from 1–4 (<10 distinct principal OTUs occurred per host specimen, S1 Table). Both the number of potential symbionts and their corresponding H’ diversity varied between sites and across host species (Fig 3). In *Idas modiolaeformis* multiple symbioses (2–4 potential symbionts) were recorded in all hosts except LD 1 and LD 3 from the Lacaze-Duthiers Canyon on sunken wood, where one symbiont only was identified (OTU 1, Fig 3). Corresponding symbiont diversity in *I*. *modiolaeformis* varied from 0–1.17 (mean = 0.38 ±0.4). By contrast, the number of potential symbionts identified per specimen in *I*. *simpsoni* never exceeded 2 OTUs (i.e. OTU 287, and either OTU 1 or OTU 3, Fig 3). Symbiont diversity in *I*. *simpsoni* gill tissues was therefore lower overall than in *I*. *modiolaeformis*, varying from 0 to 0.48 (mean = 0.10 ±0.2, Fig 3, S1 Table).

#### OTU distribution by species and site

All seven potential symbiont OTUs recorded in the current study were identified at least once in *Idas modiolaeformis*. However, two OTUs were particularly abundant, based on the number of reads recorded for each symbiont identified. Indeed, OTUs 1 and 3 respectively contributed 50% and 38% of the 46736 principal-OTU reads recorded in *I*. *modiolaeformis* (Fig 3). OTU 1 was abundant in *I*. *modiolaeformis* collected in the East Mediterranean (61–94% and 54–70% of reads ind.^-1^ from AMS and NDSF respectively), in the West Mediterranean (97–≈100% of reads ind.^-1^ from LD) and from DAR in the East Atlantic (40–79% of reads ind.^-1^). However OTU 1 was rare in *I*. *modiolaeformis* found at other Atlantic sites (GOR and MEK, only 0.04–1.2% of reads ind.^-1^, Fig 3). This OTU was also identified in an *I*. *simpsoni* specimen (LD4, Fig 3, detailed below). The second most abundant sequence, OTU 3, was recorded in at least one *I*. *modiolaeformis* specimen per site (and one *I*. *simpsoni* mussel, LD 5, Fig 3). Highest relative abundances were recorded in specimens from the East Atlantic (Fig 3, MEK: 87–97%; GOR: 86–99%; and DAR: 12–47% of reads ind.^-1^). Incidences were much reduced at all Mediterranean sites by comparison (<1–11% of reads ind.^-1^ at AMS, NDSF and LD, Fig 3). Other symbiont-related OTUs in *I*. *modiolaeformis* were much less abundant based on OTU reads (S1 Table), only once exceeding 30% of reads ind.^-1^ (OTU 2, in specimen AMS 1, Fig 3). OTU 2 contributed 2–30% and 13–20% of reads ind.^-1^ at AMS and NDSF, respectively (Fig 3; S1 Table). In addition, two SOX-related bacteria not yet recorded in this host species (OTUs 62 and 287, Fig 2) were identified in single specimens of *I*. *modiolaeformis*. The first (OTU 62, 19% of reads ind.^-1^, Fig 3) remains putative and was only found in the most symbiont-diverse specimen (NDSF 3, H’ = 1.17). The second was found at very low abundances in GOR 2 (OTU 287, <1% of reads, Fig 3). OTU 86 was found exclusively in *I*. *modiolaeformis* collected at East Atlantic sites and contributed up to 10% of reads ind.^-1^ at GOR, MEK and DAR (Fig 3). The MOX symbiont (OTU 31) occurred at the lowest number of maximum reads of any symbionts in *I*. *modiolaeformis* specimens (1% and 2% of reads ind.^-1^ at East Atlantic sites AMS 3 and NDSF 2 respectively, Fig 3).

Although 3 distinct OTUs were identified in specimens of *I*. *simpsoni*, one SOX symbiont was particularly abundant (OTU 287), regardless of whether specimens were sampled from bone remnants (SET, slightly ≤100% of reads ind.^-1^), or sunken wood (LD, 80–83% of reads ind.^-1^). The only other symbiont sequences identified in *I*. *simpsoni*, exclusively from LD, were OTUs 1 (17% of reads at LD 4, Fig 3) and 3 (<1% of reads at LD 5). These two OTUs, not yet recorded in this host species, occurred regularly in *I*. *modiolaeformis* in the current study.

### Symbiont-assemblage structure

#### Ordination analysis

**Fig 4:**
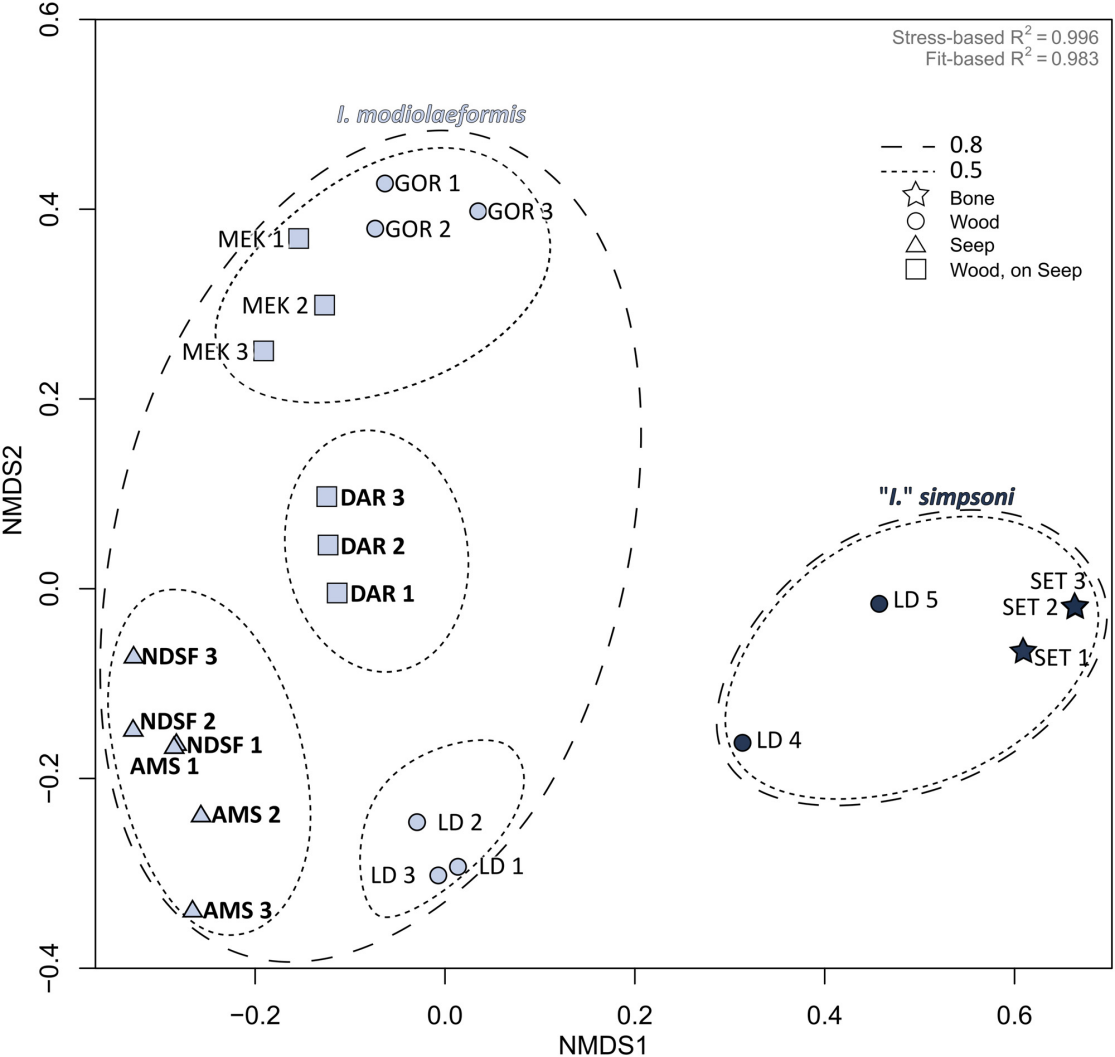
nMDS ordinance plot of bacterial assemblages in *Idas modiolaeformis* and *I*. *simpsoni*. A BC dissimilarity-matrix derived from no. reads for each OTU per host was used to plot nMDS. Symbol shapes indicate HABITAT (see legend). Light blue = *I*. *modiolaeformis*; navy = *I*. *simpsoni*. *S*uperimposed ellipses correspond to the 0.5 and 0.8 heights of a separate cluster analysis (see S1 Fig). Visibly active sites are in bold.

Non-parametric MDS ordinations, based on BC dissimilarity matrices compiled from principal-assemblage abundances (Fig 4) and symbiont-only abundances (not shown), revealed similar results. For each host species, principal bacterial compositions were more consistent within each site than across them (Fig 4, S1 Table). Both the nMDS ordination and cluster analysis, performed in tandem (S1 Fig, results superimposed on Fig 4), indicated the existence of stratified segregation across mussel specimens based on dissimilarities between their associated bacterial assemblages. Data points segregated most strongly in the plot according to host species effects (e.g. along the nMDS1 axis), delineated by the 0.8- and 0.5-height ellipses derived from cluster analysis (Fig 4). This was supported by strong, statistically significant global differences between HOST only (*n* = 2, R = 0.838, P <0.01, 999 permutations, where *n* = number of groups). Beyond the host species effect, data points also segregate in the plot according to site (Fig 4), a fact supported by statistically significant global differences among SITE groups (*n* = 7, R = 0.800, P <0.01, 999 permutations) and to a lesser extent, by HABITAT groups (*n* = 4, R = 0.508, P <0.01, 999 permutations). However, relatively low dissimilarities between the East Mediterranean sites NDSF and AMS, and between the Atlantic sites MEK and GOR resulted in these sites grouping in pairs below the 0.5-height threshold (Fig 4, threshold defined in S1 Fig). However, it was when HOST and SITE effects were combined as two levels in a single grouping factor, that the strongest statistical global differences were identified (N = 8, R = 0.965, P <0.01, 999 permutations).

With HOST and SITE still combined as two levels of a single grouping factor, SIMPER identified the dominant OTUs 1, 3, and 287 as the principal contributors to overall BC dissimilarities recorded among specimens (S2 Table). On rare occasions, OTU 2 also contributed between 18% (NDSF—DAR) and 20% (NDSF—AMS) to overall BC dissimilarities (S2 Table).

Finally, a moderate correlation (Mantel test, ρ = 0.53, P <0.01) was identified between bacterial-assemblage dissimilarities in *Idas modiolaeformis* and the physical distances separating the sites at which this species occurred (i.e. excluding Setúbal; intra-site distances were set at zero).

## Discussion

While pyrosequencing techniques are not strictly quantitative, the degree to which symbiotic OTUs dominated the reads from each of the mussels examined certainly suggest that these bacteria figured significantly in host populations. The consistency of compositions in replicate specimens further suggests these data are representative. By accounting for intra- and inter-specific variability, our dataset provides notable insight into the processes governing symbiont community compositions in deep-sea mussels.

### Host and symbiont distributions operate independently

Sampling sites were located in three oceanographic regions, where considerable distances between some sites could result in limited genetic exchange between mussels. Yet the highest host mtCOI K2P distances (2–2.4%) in *Idas modiolaeformis* were recorded within the East Atlantic (DAR), while the most common host mtCOI haplotypes of both species were shared across distinct regions, providing evidence for limited genetic differentiation in the current study. This level of genetic exchange between distinct oceanographic regions is most probably due to larval transport networks. Pelagic larval durations are thought to be long in bathymodiolins, including *I*. *modiolaeformis* [24], allowing increased periods of dispersal as veligers [40].

Of the symbiont OTUs identified, the most commonly occurring were present in all three regions (OTUs 1, 3) or both sides of the Strait of Gibraltar (OTU 287), with only the rarer OTUs being restricted to single oceanographic regions in the East Atlantic (OTU 86) and the East Mediterranean (OTUs 2, 31 and the unconfirmed symbiont 62). This suggests that much like host haplotypes, the distributions of several dominant symbiont OTUs are not restricted by the straits separating the three regions of the study. The distribution of host haplotypes and the compositions of their symbiont assemblages varied independently of one another, suggesting that larval and symbiont sources differ.

### Contrasting symbiont assemblages in each host species

Although *Idas modiolaeformis* individuals can host up to six discrete gill-associated bacteria, most appear to harbour subsets of these symbionts [7,9,12]. Five out of the six recorded symbionts of *I*. *modiolaeformis* were found in the current study, all of them Gammaproteobacteria (OTUs 1, 2, 3, 31 and 86, Fig 2). The most abundant, OTU 1, corresponded to a SOX symbiont identified in specimens from DAR (clone G-3.2, HE963019, [9]) and to one of two closely-related SOX symbionts abundant in specimens from the East Mediterranean (clone M1.17, AM402957, [7]). Exact matches for the other East-Mediterranean SOX symbiont (AM402956 [7]) were not found herein (Fig 2). OTU 2 corresponded to a bacterium related to methylotrophs, which has been recorded regularly at sub-dominant levels in *I*. *modiolaeformis* recovered from the East Mediterranean, as in the current study. OTU 3 corresponded to a clade of several bacteria within which clones G-2a.1, G-2a.2, G-2b.1 and G-2b.2 reside [9], each related to the gill-associated bacterium ‘symbiont-G’ (e.g. clone M4.36, [7]). OTU 86, found at low abundances herein at MEK, GOR and in one specimen at DAR, corresponded to a known potential symbiont related to the ‘symbiont-G’ lineage and previously described in specimens from the Gulf of Cadiz (clone G-4.1 [9]). Relative abundances of OTUs 3 and 86 in the current study resembled those reported in [9]. Although the numerical importance of symbiont-G type bacteria is high at GOR and MEK, their roles as symbionts remain unclear and they probably lack SOX and MOX capabilities [9]. Finally, a putative symbiont (OTU 62) in *I*. *modiolaeformis* may represent either an additional and rare SOX symbiont, or a local free-living strain, based on its nearest phylogenetic affinities. Two symbiotic bacteria normally common in *I*. *modiolaeformis*, the MOX symbiont (i.e. OTU 31) and the unrecorded East-Mediterranean SOX symbiont (clone M1.2 [7]), were either low in abundance or conspicuously absent. Symbiont compositions actively respond to the availability and composition of reduced fluids. Since fluids are spatially and temporally heterogeneous at many East Mediterranean seeps [41], the lack of these symbionts may simply reflect external conditions that led to their absence locally. Relatively low abundances of OTU 31 in *I*. *modiolaeformis* may also have been down-weighted by two base mismatches identified *post-priori* in the V5-V6 region of the universal GS-FLX F-primer [42], even though these did not exclude the identification of this bacterium entirely.

In stark contrast, when *I*. *simpsoni* is the only chemosymbiotic bathymodioline present at a sampling site, a single SOX chemolithoautotroph dominates its gills (OTU 287, Fig 2) to such an extent that in two specimens, no other bacterial OTU occurred at >1% of total OTU reads (SET 2–3). OTU 287 was found almost exclusively in *I*. *simpsoni* and was distinct from OTU 1, the prevalent SOX symbiont in *I*. *modiolaeformis*. The partial 16S rRNA sequence for OTU 287 was identical to the SOX symbiont identified in other SET specimens analysed by [14] and almost identical to the symbiont identified in host mussels from seep-like habitats in the Marmara Sea, later confirmed to be *I*. *simpsoni* [6]. The only other symbionts identified in *I*. *simpsoni* in the current study were OTUs 1 and 3, identified in *I*. *modiolaeformis* as the main SOX symbiont and the ubiquitous ‘symbiont-G’-related bacterium respectively. However these were both less abundant in *I*. *simpsoni* by comparison. Overall, *I*. *simpsoni* evidently displays a high degree of fidelity to its principal SOX symbiont (OTU287 herein).

Symbiont diversity was consistently higher in *I*. *modiolaeformis* than in *I*. *simpsoni*, with little compositional overlap. The highest BC dissimilarities were thus identified in relation to host. Beyond the host effect, *I*. *modiolaeformis* also saw differences among sites. Assemblages were dominated by SOX- and methylotrophs at seeps in the East Mediterranean (e.g. OTUs 1 and 2), by symbiont-G-related bacteria in the East Atlantic (i.e. OTUs 3 and 86). Assemblages recorded in specimens from the geographically intermediate, active seep-site DAR, involved both SOX and Symbiont-G-related bacteria. In both host species, assemblages at LD were dominated by one type of SOX symbiont only (OTU 1 in *I*. *modiolaeformis* and OTU 287 in *I*. *simpsoni*). Differences in relation to collection site (regardless of species) were significant, based on the ANOSIM performed with site as the grouping factor. The highest ANOSIM R values however, were obtained when host and site were used together as two levels in a single grouping factor, allowing their collective influence to be tested. Overall, symbiont assemblages depend mainly on host identity, with environmental factors playing an important secondary role in *I*. *modiolaeformis*.

### Potential factors influencing symbiont community composition

Symbiont assemblages of *Idas modiolaeformis* displayed distinct patterns both regionally and between several neighbouring sites, varying independently of host mtCOI-haplotype. Previous studies have demonstrated the absence of bacteria in male and female gametes [24], and in post-larvae of *I*. *modiolaeformis* [43], with symbiont acquisition following a brief period of juvenile growth. The diverse symbiont assemblages observed in *I*. *modiolaeformis* thus depend mostly on the local availability of symbionts at settlement sites, and the capacity of the host to accommodate them. In the current study, symbioses in *I*. *modiolaeformis* varied from being metabolically diverse in specimens from East Mediterranean seep regions, which are characterised by complex reduced fluids containing methane and sulphides [41,44], to being exclusively thiotrophic in specimens on decomposing wood at LD, where conditions may be more sulphidic [45,46]. That said, due to potential sequencing biases against OTU 31 in the current study, the presence of MOX symbionts at very low densities at LD cannot be ruled out completely. In the East Atlantic at GOR and MEK, symbiont assemblages in *I*. *modiolaeformis* were very different to those in the Mediterranean, due to the high abundances of OTUs 3 and 86. Since the symbiotic roles of the symbiont-G type bacteria remain unknown, it is not possible to determine what governs their particularly high abundance at these two sites compared to others, other than the observation that seep activity was comparatively non-existent or lower than in DAR [9]. This reasserts the need to better understand the potential roles of the numerous symbiont-G-type bacteria engaged in symbioses with *I*. *modiolaeformis*. Certainly the presence of SOX symbionts in specimens at DAR suggests that this site was not only seeping actively (methane bubbling was observed during the deployment of the CHEMECOLI) but that fluids contained sulphides.

Much like *I*. *modiolaeformis*, *I*. *simpsoni* appears to lack detectable symbionts as post-larvae, with local symbiont acquisition first occurring immediately following the onset of juvenile growth [47]. This suggests that the associated SOX symbiont (OTU 287) is ubiquitous across a diverse array of habitats in different oceanographic regions. *I*. *simpsoni* does not appear to display a capacity for diverse, site-specific associations, even in seep-associated settings such as the Marmara Sea [15] where both methane and sulphide production are known to occur [48,49]. Since local availability is unlikely to be restricted to a single SOX bacterium, low symbiont diversity in *I*. *simpsoni* must depend upon other factors which have yet to be identified.

It is intriguing that despite similar post-settlement acquisition modes, the two species display quite distinct levels of host-symbiont fidelity. One way in which access to locally abundant symbiont candidates might be enhanced, is through direct interaction with conspecifics. Observations made during sample collection indicate that small-scale distribution patterns for each species differ markedly, independent of habitat. While distributions of *I*. *modiolaeformis* were discrete and patchy on seep-associated carbonate crusts and on plant debris, *I*. *simpsoni* specimens were gregarious, forming dense clumps on wood (LD) and bone (SET). Dense aggregations such as these are a feature of larger Bathymodiolinae at hydrothermal vents and cold seeps, in which symbioses are also typically restricted to one or two bacterial phylotypes [1]. Under these circumstances, conspecific symbiont transmission (a form of ‘lateral’, host-to-host transmission) would occur more readily and rapidly between *I*. *simpsoni* individuals than in *I*. *modiolaeformis*, and may explain the less diverse assemblages seen in the former. Lateral transmission of this sort, if it can be shown to occur interspecifically, would help to explain the appearance of the atypical symbionts observed at low abundances in both species. This also provides a potential avenue by which novel symbionts might be acquired.

## Conclusions

The low symbiont diversity seen in *Idas simpsoni* is typical of most Bathymodiolinae investigated to date [1]. In most *Idas* spp. from organic falls, SOX symbionts dominate symbioses [13,50,51]. Given the prolific success of Bathymodiolinae at many reducing habitats, this strategy must provide fitness advantages for survival in chemosynthesis-based systems, provided that the availability of sulphides meets their requirements. By forming dense clumps, the presence of conspecifics likely augments the pool of bacteria available to proximal hosts, allowing earlier, more rapid acquisition of symbionts following settlement. High symbiont diversity has on the other hand been proposed as an adaptive trait in *I*. *modiolaeformis*, allowing the species to take advantage of diverse energetic resources. In other words, rather than engaging in a rigid, targeted symbiosis, *I*. *modiolaeformis* employs a more flexible, generalist strategy. However, the fact that populations never reach the densities characteristic of mussels with highly conservative symbioses suggests that this flexibility may come at an energetic cost, possibly in the form of lower growth rates or reduced reproductive output. Overall the current study demonstrates that two superficially-similar, symbiont-bearing mussels can display markedly distinct yet effective strategies. This emphasizes the need to investigate the full diversity of symbioses within this group of chemosymbiotic metazoans.

## Supporting Information

S1 Fig Cluster analysis of bacterial assemblages in *Idas modiolaeformis* and *I*. *simpsoni*. (PNG)

S1 Table Principal bacterial OTUs found in the gill tissue of mussels in the current study. Displayed, is the number of reads for the full complement of principal OTUs (i.e. those which contributed to >1% of total abundances in any one mussel). Symbiotic OTUs are listed first (total of 7). Accession numbers attributed to sequences from the current study are accompanied by the accession number for the nearest hit definition identified during initial BLAST (May 2015).
(DOCX)

S2 Table The accumulative percentage contributions (up to +70%) of individual OTUs to overall Bray-Curtis dissimilarities between species and site levels collectively. (DOCX)
