# Supplementary figures and images for: Fickle or Faithful: The Roles of Host and Environmental Context in Determining Symbiont Composition in Two Bathymodioline Mussels
Source: PLoS One. 2015 Dec 28;10(12):e0144307. doi: 10.1371/journal.pone.0144307 (PMC4692436; doi:10.1371/journal.pone.0144307)

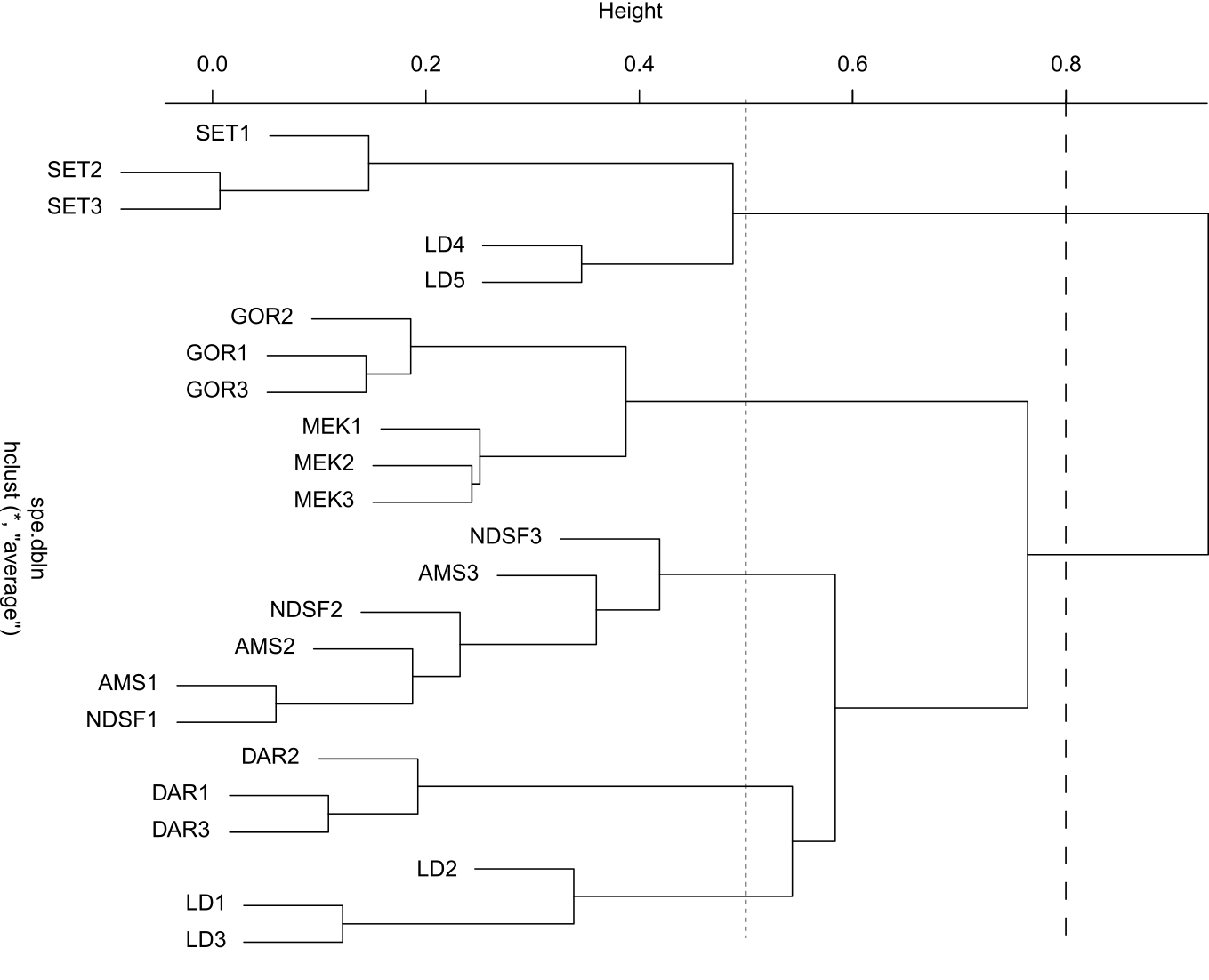

Supplement: S1 Fig — (PNG) [file pone.0144307.s001.png]
