# Supplementary material for: Fickle or Faithful: The Roles of Host and Environmental Context in Determining Symbiont Composition in Two Bathymodioline Mussels
Source: PLoS One. 2015 Dec 28;10(12):e0144307. doi: 10.1371/journal.pone.0144307 (PMC4692436; doi:10.1371/journal.pone.0144307)
Supplement: S1 Table — Displayed, is the number of reads for the full complement of principal OTUs (i.e. those which contributed to >1% of total abundances in any one mussel). Symbiotic OTUs are listed first (total of 7). Accession numbers attributed to sequences from the current study are accompanied by the accession number for the nearest hit definition identified during initial BLAST (May 2015). (DOCX) [file pone.0144307.s002.docx]

|  | *Idas modiolaeformis*  (reads OTU^-1^) | | | | | | | | | | | | | | | | | | “*Idas*” *simpsoni*  (reads OTU^-1^) | | | | |  |  |  |
| --- | --- | --- | --- | --- | --- | --- | --- | --- | --- | --- | --- | --- | --- | --- | --- | --- | --- | --- | --- | --- | --- | --- | --- | --- | --- | --- |
| OTU name | AMS 1 | AMS 2 | AMS 3 | NDSF 1 | NDSF 2 | NDSF 3 | DAR 1 | DAR 2 | DAR 3 | MEK 1 | MEK 2 | MEK 3 | GOR 1 | GOR 2 | GOR 3 | LD 3 | LD 4 | LD 5 | LD 1 | LD 2 | SET 1 | SET 2 | SET 3 | Accession  (this study) | Accession  (hit definition) | **ID match (%)** |
| OTU 1 | 2065 | 1617 | 2751 | 1562 | 951 | 908 | 1525 | 1583 | 653 | 4 | 6 | 64 |  | 1 |  | 3066 | 3960 | 2900 | 488 |  |  |  |  | KT216459 | HM441251 | 99 |
| OTU 2 | 1030 | 193 | 63 | 463 | 253 | 229 |  |  |  |  |  |  |  |  |  |  |  |  |  |  |  |  |  | KT216460 | AM402960 | 99 |
| OTU 3 | 79 | 3 |  | 85 | 138 | 187 | 235 | 1363 | 774 | 1525 | 1490 | 4802 | 2335 | 2347 | 2525 |  | 4 |  |  | 4 |  |  |  | KT216461 | JN233229 | 99 |
| OTU 31 |  |  | 42 |  | 30 |  |  |  |  |  |  |  |  |  |  |  |  |  |  |  |  |  |  | KT216465 | GU584415 | 99 |
| OTU 62 |  |  |  |  |  | 316 |  |  |  |  |  |  |  |  |  |  |  |  |  |  |  |  |  | KT216468 | FM246510 | 99 |
| OTU 86 |  |  |  |  |  |  |  |  | 18 | 135 | 42 | 150 | 276 | 16 | 19 |  |  |  |  |  |  |  |  | KT216471 | HE963023 | 99 |
| OTU 287 |  |  |  |  |  |  |  |  |  |  |  |  |  |  | 2 |  |  |  | 2157 | 1348 | 4225 | 3373 | 3015 | KT216476 | HE814577 | 99 |
| OTU 4 | 143 | 72 | 39 | 91 | 32 |  |  |  |  |  |  |  |  |  |  |  |  |  |  |  |  |  |  | KT216462 | GU584307 | 96 |
| OTU 5 | 24 |  |  | 6 | 16 | 24 |  |  |  |  |  |  |  |  |  |  |  |  |  |  |  |  |  | KT216463 | EU287240 | 94 |
| OTU 7 | 27 | 3 |  | 15 | 18 |  |  |  |  |  |  |  |  |  |  |  |  |  |  |  |  |  |  | KT216464 | GU235132 | 94 |
| OTU 34 |  |  | 47 |  | 4 |  |  |  |  |  |  |  |  |  |  |  |  |  |  |  |  |  |  | KT216466 | FM246510 | 95 |
| OTU 56 |  |  |  |  | 12 |  | 96 |  | 105 |  |  |  |  |  |  |  |  |  |  |  |  |  |  | KT216467 | GU235078 | 97 |
| OTU 65 |  |  |  |  |  | 22 |  |  |  |  |  |  |  |  |  |  |  |  |  |  |  |  |  | KT216469 | GU235132 | 93 |
| OTU 73 |  |  |  |  |  |  | 82 | 54 | 85 |  |  |  |  |  |  |  |  |  |  |  |  |  |  | KT216470 | GU235132 | 97 |
| OTU 115 |  |  |  |  |  |  |  |  |  | 46 | 3 | 394 |  |  |  |  |  |  |  |  |  |  |  | KT216472 | EU287124 | 97 |
| OTU 117 |  |  |  |  |  |  |  |  |  | 34 |  |  |  |  |  |  |  |  |  |  |  |  |  | KT216473 | JQ337642 | 92 |
| OTU 130 |  |  |  |  |  |  |  |  |  | 7 |  |  | 9 | 7 | 32 |  |  |  |  |  |  |  |  | KT216474 | AB424899 | 90 |
| OTU 239 |  |  |  |  |  |  |  |  |  |  |  |  | 79 | 5 | 189 |  |  |  |  |  |  |  |  | KT216475 | JN424328 | 91 |
| OTU 310 |  |  |  |  |  |  |  |  |  |  |  |  |  |  |  | 6 |  | 1 | 41 | 155 | 11 |  |  | KT216477 | JQ347404 | 96 |
| OTU 337 |  |  |  |  |  |  |  |  |  |  |  |  |  |  |  |  | 119 |  |  |  |  |  |  | KT216478 | EU236385 | 95 |
| OTU 339 |  |  |  |  |  |  |  |  |  |  |  |  |  |  |  |  | 1 | 2 | 8 | 25 |  |  |  | KT216479 | KJ814567 | 97 |
| OTU 390 |  |  |  |  |  |  |  |  |  |  |  |  |  |  |  |  |  |  | 7 | 59 |  |  |  | KT216480 | KJ814570 | 99 |
| OTU 440 |  |  |  |  |  |  |  |  |  |  |  |  |  |  |  |  |  |  |  | 30 |  |  |  | KT216481 | NR116611 | 97 |
| **OTUs host^-1^** | **6** | **5** | **5** | **6** | **9** | **6** | **4** | **3** | **5** | **6** | **4** | **4** | **4** | **5** | **5** | **2** | **4** | **3** | **5** | **6** | **2** | **1** | **1** |  |  |  |
| Reads host^-1^ | **3368** | **1888** | **2942** | **2222** | **1454** | **1686** | **1938** | **3000** | **1635** | **1751** | **1541** | **5410** | **2699** | **2376** | **2767** | **3072** | **4084** | **2903** | **2701** | **1621** | **4236** | **3373** | **3015** |  |  |  |
| % symb. | **94.2** | **96.0** | **97.1** | **95.0** | **94.4** | **97.3** | **90.8** | **98.2** | **88.4** | **95.0** | **99.8** | **92.7** | **96.7** | **99.5** | **92.0** | **99.8** | **97.1** | **99.9** | **97.9** | **83.4** | **99.7** | **100.0** | **100.0** |  |  |  |
