# Supplementary material for: Fickle or Faithful: The Roles of Host and Environmental Context in Determining Symbiont Composition in Two Bathymodioline Mussels
Source: PLoS One. 2015 Dec 28;10(12):e0144307. doi: 10.1371/journal.pone.0144307 (PMC4692436; doi:10.1371/journal.pone.0144307)
Supplement: S2 Table — (DOCX) [file pone.0144307.s003.docx]

| **NDSF  (I. mod)** | **OTU1** | **OTU2** |  |  | |  |  |  |  |  |  |  |  |  |  |  |
| --- | --- | --- | --- | --- | --- | --- | --- | --- | --- | --- | --- | --- | --- | --- | --- | --- |
|  | 0.59 | 0.79 |  |  | |  |  |  |  |  |  |  |  |  |  |  |
| **DAR  (I. mod)** | **OTU1** | **OTU3** | **OTU3** | **OTU1** | | **OTU2** |  |  |  |  |  |  |  |  |  |  |
|  | 0.39 | 0.70 | 0.35 | 0.62 | | 0.80 |  |  |  |  |  |  |  |  |  |  |
| **MEK  (I. mod)** | **OTU3** | **OTU1** | **OTU3** | | **OTU1** | | **OTU3** | **OTU1** |  |  |  |  |  |  |  |  |
|  | 0.43 | 0.85 | 0.52 | | 0.81 | | 0.48 | 0.88 |  |  |  |  |  |  |  |  |
| **GOR  (I. mod)** | **OTU3** | **OTU1** | **OTU3** | | **OTU1** | | **OTU3** | **OTU1** | **OTU3** | |  |  |  |  |  |  |
|  | 0.46 | 0.86 | 0.55 | | 0.83 | | 0.51 | 0.89 | 0.78 | |  |  |  |  |  |  |
| **SET  (I. simp)** | **OTU287** | **OTU1** | **OTU287** | | **OTU1** | | **OTU287** | **OTU1** | **OTU287** | **OTU3** | **OTU287** | **OTU3** |  |  |  |  |
|  | 0.57 | 0.91 | 0.66 | | 0.88 | | 0.62 | 0.84 | 0.58 | 0.96 | 0.57 | 0.97 |  |  |  |  |
| **LD  (I. mod)** | **OTU1** | **OTU2** | **OTU1** | | | | **OTU1** | **OTU3** | **OTU1** | **OTU3** | **OTU1** | **OTU3** | **OTU287** | **OTU1** |  |  |
|  | 0.66 | 0.89 | 0.76 | | | | 0.68 | 0.94 | 0.57 | 0.95 | 0.55 | 0.96 | 0.51 | 0.99 |  |  |
| **LD  (I. simp)** | **OTU1** | **OTU287** | **OTU287** | | **OTU1** | | **OTU287** | **OTU1** | **OTU3** | **OTU287** | **OTU3** | **OTU287** | **OTU287** | | **OTU1** | **OTU287** |
|  | 0.44 | 0.83 | 0.495707 | | 0.76094 | | 0.45 | 0.71 | 0.48 | 0.86 | 0.51 | 0.87 | 0.82 | | 0.61 | 0.96 |
|  | **AMS (I. mod)** | | **NDSF (I. mod)** | | | | **DAR (I. mod)** | | **MEK (I. mod)** | | **GOR (I. mod)** | | **SET (I. simp)** | | **LD (I. mod)** | |
